# Demoralization level fluctuated at various time frame of radiotherapy for patients with different cancers: a longitudinal observational study

**DOI:** 10.1186/s12904-022-01033-z

**Published:** 2022-08-10

**Authors:** Pei-Ling Tang, Huey-Shyan Lin, Hsiu-Hung Wang, Li-Yu Hu, Fan-Hao Chou

**Affiliations:** 1 Research Center of Medical Informatics, Kaohsiung Veterans General Hospital, 386, Dazhong 1st Rd., Zuoying Dist., Kaohsiung City, 813414 Taiwan (ROC); 2 School of Nursing, Fooyin University, 151 Jinxue Rd., Daliao Dist., Kaohsiung City, 83102 Taiwan (ROC); 3 School of Nursing, Kaohsiung Medical University, 100, Shin-Chuan 1st Road, Sanmin Dist., Kaohsiung City, 80708 Taiwan (ROC); 4 Department of Health-Business Administration, Fooyin University, 151 Jinxue Rd., Daliao Dist., Kaohsiung City, 83102 Taiwan (ROC); 5 Department of Psychiatry, Taipei Veterans General Hospital, 201, Sec.2, ShihPai Rd., Beitou District., Taipei City, 11217 Taiwan (ROC); 6 Division of Psychiatry, National Yang-Ming University, 155, Sec.2, Linong St., Beitou District, Taipei City, 11221 Taiwan (ROC)

**Keywords:** Cancer, Demoralization, Psycho-oncology, Radiotherapy

## Abstract

**Background:**

Demoralization is a psychological response that is frequently observed in patients with cancer or advanced diseases. It is affected by national characteristics, culture, disease characteristics and general conditions of the patient such as individual cultural features, nature of stress, personal expression preferences and social behavior. Compared with the results of previous studies on demoralization syndrome, patients with cancer in Taiwan exhibit a higher prevalence of demoralization. We aimed to investigate the prevalence of high demoralization and the changes in the level of demoralization in cancer patients during radiotherapy to explore the associated factors and the contributing factors to the high level of demoralization.

**Methods:**

We used the Demoralization Scale-Mandarin Version to evaluate the demoralization level at six-time points in patients admitted for radiotherapy in a 3-month observational period. 101 patients allocated to three groups by cancer region completed the study. We applied the generalized estimating equation (GEE) to analyze the changes in the demoralization level among the three groups. The variables associated with the changes in the demoralization level were also investigated.

**Results:**

In the analysis using univariate GEE, only patients in the chest and breast group exhibited significant changes at two different time points. The results obtained using multivariate GEE revealed that sociodemographic variables, stage of disease and use of surgery or chemotherapy had no impact on the changes in demoralization across three months.

**Conclusion:**

The demoralization level certainly fluctuated in an extremely high range. The higher prevalence of demoralized patients may indicate that if medical staff neglect the importance of demoralization, demoralized patients with cancer may not receive appropriate care.

## Background

Demoralization syndrome is a common clinical presentation in patients at the end of life [1, 2]. The core concept of demoralization is the loss of purpose and meaning in a patient’s life [3]. Aside from the philosophy of existentialism, the traditional psychological theory of drive implies that people instinctively strive to live their lives, particularly in the critical moment between life and death [4]. However, demoralized patients who have lost the sense of meaningfulness of life present symptoms such as hopelessness and helplessness due to the enormous and persistent existential distress they face [1]. In 2001, Kissane et al. [5] investigated cancer patients receiving palliative care and reported that the demoralization syndrome has specific symptoms that could be identified clinically. Another study provided further evidence, emphasizing the importance of the concept of demoralization, particularly in the field of palliative care [6]. Moreover, in 2004, they proposed the demoralization scale (DS), which is a reliable and validated measure of demoralization [7]. Since its publication, the DS has been validated by several studies [8, 9] and has been used in studies investigating demoralization, facilitating the identification of patients with demoralization syndrome [10]. Moreover, the DS has also been used as a diagnostic tool, particularly for differentiating between demoralization and depression [11].

Therapeutic complexity increases the psychologic burden of patients with cancer, leading patients to experience feelings such as anxiety, depression, anxiety of death, demoralization and ineffective coping, and whether or not the patients accept the disease and feelings and continue to live their lives may affect the patients’ prognosis, quality of life, and even suicide and death rates [12]. Demoralization is different from depressive disorder in terms of clinical impact. It is suggested that depressive patients tend to perceive that the source of distress is internal to them and often lack motivation, while in contrast, demoralized patients perceive that the source of distress is external to them and frequently present with uncertainty about the direction their actions should follow, implying that their motivation is intact [1]. Multiple studies have shown that demoralization is more associated with psychological state rather than the physical condition [12–14]. Furthermore, for clinicians or medical staff devoted to caring for patients with cancer, patients under hospice care, or patients with chronic psychosis, the most important concern is not only to differentiate demoralized patients from depressive ones, but also to provide appropriate and efficient treatment strategies for patients with a high demoralization level [1]. Numerous studies have demonstrated that, for patients with depression, biological interventions such as antidepressant use are helpful, and for demoralized patients without depression, psychotherapy or other psychosocial approaches are the most effective intervention choices [1, 15, 16].

Notably, demoralization may be affected by national characteristics, culture, disease characteristics and general conditions of the patient such as individual cultural features, nature of stress, personal expression preferences and social behavior [17]. Compared with the results of previous studies on the prevalence of highly demoralized patients with cancer in other countries, patients with cancer in Taiwan exhibited a higher prevalence of demoralization syndrome [9, 10, 18]. According to our review of the relevant literature, studies on demoralization among patients with cancer in Taiwan [10] may not be easily generalized due to several limitations, such as a cross-sectional design, lack of information on the subtypes of cancer and cancer stages, and different time points in cancer treatment processes. Therefore, in the current study, we used a longitudinal design and considered many possible associated factors. We aimed to investigate the prevalence of high demoralization and the changes in the level of demoralization in cancer patients during radiotherapy (RT) to explore the associated factors and the contributing factors to the high level of demoralization.

### Conceptual framework

Demoralization syndrome has become increasingly recognized as a challenge to providing patients with grave diseases with appropriate care as they typically lose hope and self-esteem and feel helpless and incompetent [5, 17, 19]. It’s been estimated that as many as almost 29% of patients with cancer present signs of demoralization, and understanding the syndrome is critical to providing appropriate and effective care to patients with it [10]. Current cancer treatments primarily involve the use of surgery, chemotherapy, and RT [20]. More than half of all cancer patients who are treated for cancer require RT, and the side effects of RT depend on the radiated site [20]. During the RT stage of cancer treatment, patients may experience impaired immune system functions, the occurrence of comorbidity, and the side effects of cancer treatment. Because of the side effects of RT generally occur from the time RT is started until 3 months after completion of the RT [9, 10], the body composition changes of patients with cancer were measured at six time points during this period – from the time they received RT to 3 months after completion of RT. We formulated this study to investigate the development of demoralization among patients with various cancers at various time points of RT. By exploring change in the demoralization level of patients and analyzing potential factors associated with the change, we hope to provide constructive suggestions to offering appropriate care to patients with cancer at risk of demoralization.

### Methods

This was a longitudinal study with a total of 121 patients recruited between January 1, 2014 and December 31, 2014. We arranged an interview with the participants to assist them in completing the Demoralization Scale-Mandarin Version (DS-MV) [8], which is a self-report questionnaire for defining high demoralization and evaluating the changes in the demoralization level at six different time points in around 6 months. Participants were arranged for an interview to help the participants receive the body composition measure at six-time points, including before starting RT, the second and fourth weeks after the first interview, the end of RT and the first and third months after RT was completed. In addition, other demographic data and the information on the participant’s physical conditions were considered, particularly those related to cancer therapy, including affected regions of cancer, cancer stages, and treatment strategies for cancer.

The inclusion criteria in our study were as follows: (1) Patients who were diagnosed with malignancy for the first time and had no previous cancer history; (2) patients who were indicated to receive RT; (3) age more than 18 years; (4) no cognitive deficit or ability to communicate with researchers; and (5) RT performed at the outpatient setting. Non-inclusion criteria were as follows: (1) RT treatment goal as providing palliative care; (2) patients with relapsed cancer; and (3) patients who were determined to be unsuitable for participation by the attending physicians because of poor physical conditions.

During the observation period, four participants interrupted their RT treatment courses, nine participants stated that they were not willing to continue, three participants died, and the data collected from four participants were removed because of the erroneous information. The missing data rate in the study was 16.52%. Finally, 101 participants completed the study for all six time points. Subsequently, we classified the 101 participants into three groups based on the affected regions of cancer: (1) head and neck; (2) chest and breast; and (3) abdominal and pelvic groups.

The measurement of the demoralization level was based on the total scores of the DS-MV, which was translated into Mandarin in 2008 with a certificate. Based on the guidelines of Kissane et al. [5], high demoralization was defined as a DS-MV score of more than 30. The Cronbach’s alpha for individual items ranged from 0.63 to 0.88, which indicated that the DS-MV is a valid and reliable questionnaire for Taiwanese patients with cancer. In addition, in 2010, Hung et al. [8] proved that the Cronbach’s alpha value of the internal consistency of the scale was 0.92.

### Ethics statement

This study was approved by the Institutional Review Board of Kaohsiung Veterans General Hospital (VGHKS13-CT11-05). We recruited volunteers by placing posters outside the radiation oncology clinics and in the radiation oncology wards. The protocol contents were explained clearly to every patient by the principal investigator, and all patients were informed that if they were not willing to continue the study, they could withdraw from the study anytime. Informed consent was obtained from all individual participants recruited in the study. All methods were performed in accordance with the relevant guidelines and regulations (Declaration of Helsinki).

### Statistical analysis

In the study, sample size calculation was done using the G power 3.1.9.2 program, repeated measures was employed and the following settings were applied: type *I* error, *α* = 0.05; test power, (1-*β*) power = 0.8; two-tailed test; 95% confidence interval; recommended medium effect size = 0.25; six repetitions, and number of group = 1. The calculations showed that at least 19 patients were required for each cancer site. Strauss et al. estimated the patient loss rate on RT to be 13% [21]. This indicated that the minimum number of patients required for this study was 66. This study used a univariate generalized estimating equation (GEE) analysis, which was performed to explore the association between the changes in the demoralization level at different time points among patients with cancer in each group. Also, both the demographically descriptive statistics such as percentage, mean, standard error, and the analytical statistics including generalized estimation equation in the current study were registered and analyzed with SPSS statistical software for Windows, Version 20 (IBM, Armonk, NY, USA). Statistical significance was considered when *p* < 0.05.

## Results

Of the 122 recruited patients, 83% (57 men and 44 women) completed the study. For the 101 completers, the mean age was 52.8 years (standard deviation [SD] = 11.1, ranging from 18 to 82). The participants were classified into three groups according to cancer regions: 32 (31.7%) patients had head and neck cancer, 45 (44.6%) patients had cancer in the chest and breast region, and 24 (23.8%) patients had cancer in the abdominal and pelvic region. The difference between the three groups was that the chest and breast cancer group had the highest female to male ratio (33 women 12 men), more younger patients, and patients with lower cancer stages (the number of different cancer stages in patients within the three groups are presented as the sum of stage 1 and stage 2 vs. stage 3 and stage 4: 12 vs. 20 [head and neck], 33 vs. 12 [chest and breast], and 8 vs. 16 [abdominal and pelvic]). Otherwise, all the participants with cancer appeared to have several similar characters such as low educational level and being married and living with their family (Table 1).

**Table 1:** Participants characteristics (*N* = 101)

Variable	N (%)	Head and neck	Chest and breast	Abdominal and pelvic
**Sex**				
Male	57 (56.43)	29	12	16
Female	44 (43.57)	3	33	8
**Age**				
< 50	42 (41.58)	10	23	9
≥ 50	59 (58.42)	22	22	15
**Highest level of education**				
High school or low	64 (63.36)	22	27	15
University or higher	37 (36.64)	10	18	9
**Marital status**				
Single	24 (23.76)	7	10	7
Married	77 (76.24)	25	35	17
**Main source of income**				
Oneself	68 (67.33)	25	23	20
Family or child	33 (32.67)	7	22	4
**Currently working**				
No	43 (42.57)	13	24	6
Yes	58 (57.43)	19	21	18
**Residential situation**				
Living alone	8 (7.92)	2	5	1
Living with family	93 (92.08)	30	40	23
**Cancer stage**				
1	35 (34.65)	9	22	4
2	18 (17.82)	3	11	4
3	30 (29.70)	8	7	15
4	18 (17.82)	12	5	1
**Operation**				
No	34 (33.66)	16	9	9
Yes	67 (66.34)	16	36	15
**Chemotherapy**				
No	44 (43.56)	20	13	11
Yes	57 (56.44)	12	32	13

**Fig. 1:**
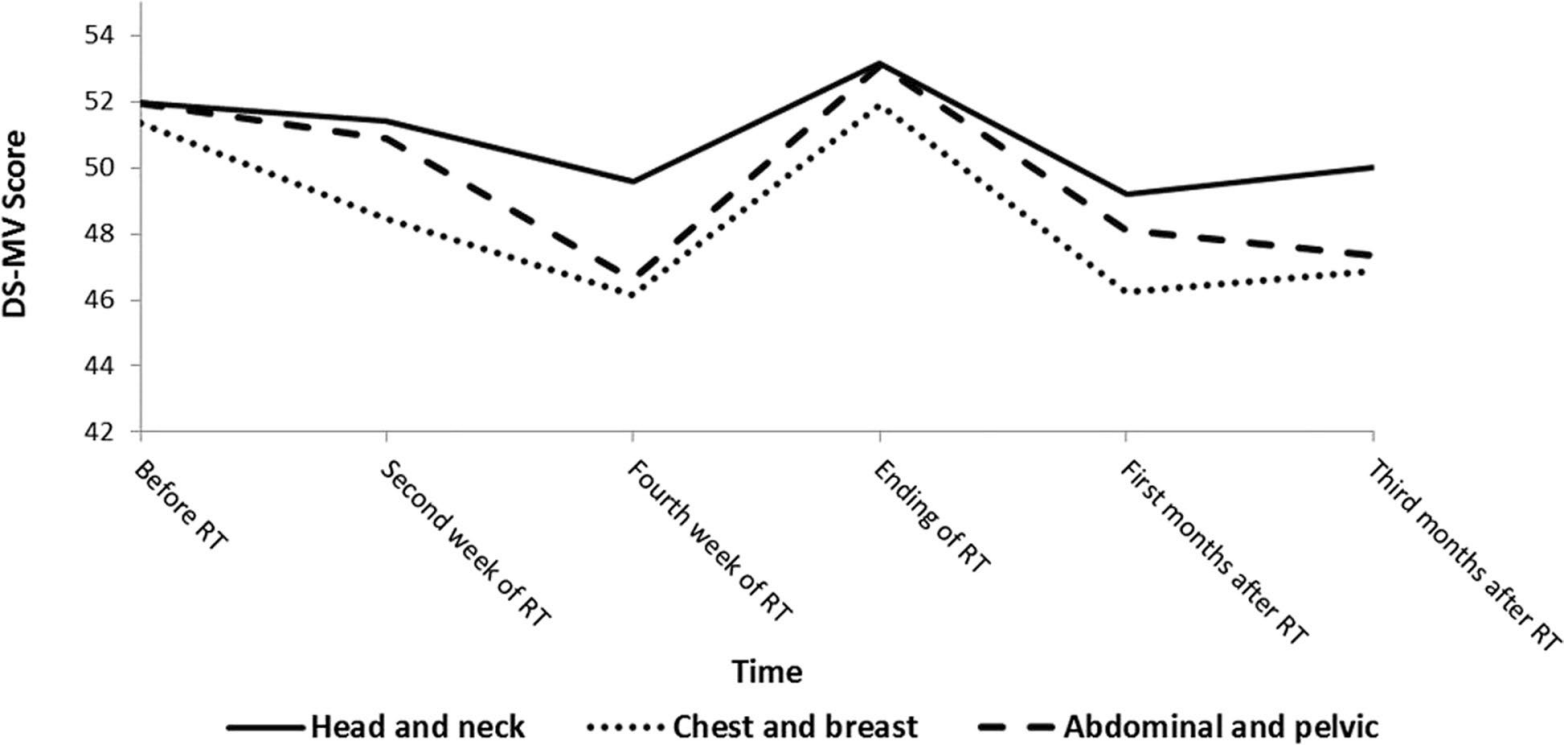
Line graphs for changes in demoralization in patients with cancer receiving radiotherapy

The DS-MV mean scores are presented according to not only different cancer regions but also six-time points during the 3-month observation period. Figure 1 presents the DS-MV scores of the patients during the entire study period by group. DS-MV score ranges among patients were as follows: head and neck cancer group: 49.18–53.17, chest and breast cancer group: 46.14–51.89, and abdominal and pelvic cancer group: 46.60–53.10.

Subsequently, we collected the demographic variables that might influence the fluctuation in the demoralization level and applied multivariate GEE analysis to investigate which variables were statistically significant confounding factors. The results showed that only patients with cancer in the chest and breast group exhibited statistically significant change at two weeks after RT and one month after completing RT (*β* =  − 2.87, *p* = 0.0447, and *β* =  − 5.09, *p* = 0.0136; Table 2). None of the variables reached statistical significance (Table 3).

**Table 2:** Generalized estimating equation for change in demoralization among cancer patients with radiotherapy

Variable	Head and neck					Chest and breast				Abdominal and pelvic		
	*β*	Standard Error	*Z* score	*P* value	*β*	Standard Error	*Z* score	*P* value	*β*	Standard Error	*Z* score	*P* value
Intercept	51.96	2.43	21.34	< 0.0001	51.33	2.17	23.67	< 0.0001	51.93	2.40	21.66	< 0.0001
Time												
2^nd^ week of RT	-0.53	2.23	-0.24	0.8117	-2.87	1.43	-2.01	0.0447	-1.05	2.30	-0.46	0.6473
4^th^ week of RT	-2.36	3.71	-0.63	0.5255	-5.19	3.72	-1.40	0.1630	-5.33	3.12	-1.71	0.0881
The end of RT	1.21	3.20	0.38	0.7056	0.56	2.55	0.22	0.8261	1.17	3.84	0.30	0.7606
1^st^ month after completing RT	-2.78	2.71	-1.03	0.3041	-5.09	2.06	-2.47	0.0136	-3.83	4.13	-0.93	0.3541
3^rd^ months after completing RT	-1.96	3.23	-0.61	0.5448	-4.48	2.88	-1.56	0.1199	-4.56	3.75	-1.22	0.2239
Before RT	Reference				Reference				Reference			

*RT* Radiotherapy

**Table 3:** Generalized estimating equation for change in demoralization among patients with chest and breast cancer with radiotherapy

Variable	Chest and breast			
	*β*	Standard Error	*Z* score	*P* value
Intercept	58.05	7.09	8.19	< 0.0001
Time				
2^nd^ week of RT	-2.93	1.44	-2.04	0.0418
4^th^ week of RT	-5.01	3.72	-1.35	0.1781
The end of RT	0.71	2.59	0.27	0.7846
1^st^ month after completing RT	-4.87	1.96	-2.48	0.0131
3^rd^ months after completing RT	-4.27	2.81	-1.52	0.1278
Before RT	Ref			
Sex				
Male vs. Female	-1.54	4.78	-0.32	0.7473
Age				
≥ 50 vs. < 50	-0.40	1.99	-0.20	0.8407
Highest level of education				
High school or low vs. University or higher	0.70	2.25	0.31	0.7542
Marital status				
Single vs. Married	0.41	3.80	0.11	0.9140
Main source of income				
Oneself vs. Other	-1.11	1.76	-0.63	0.5266
Occupation				
No vs. Yes	0.03	1.92	0.02	0.9879
Cancer stage				
2 vs. 1	-1.96	2.52	-0.78	0.4370
3 vs. 1	2.09	3.10	0.67	0.5004
4 vs. 1	-2.66	3.44	-0.77	0.4388
Operation				
No vs. Yes	-1.26	4.77	-0.27	0.7909
Chemotherapy				
No vs. Yes	-0.15	2.27	-0.07	0.9474

*RT* Radiotherapy. Adjusted for age, sex, highest level of education, marital status, main source of income, occupation, residential situation, cancer stage, operation, chemotherapy

## Discussion

As far as we know, the current work is the first trial in Taiwan to use a longitudinal study design to explore the prevalence of high demoralization and the demoralization level changes according to the DS-MV scores on cancer patients hospitalized for RT. Also, the information on the cancer regions and cancer stages were collected for more in-depth analysis.

There were three major findings in the study. Firstly, nearly all participants revealed unusually higher demoralization levels than the level defined by DS-MV to be highly demoralized, 30 [18], regardless of cancer region, cancer stage or cancer treatment strategy. As shown in Table 2, the ranges of the DS-MV mean scores among the three cancer region groups during the study period were 49.18–53.17 in the group of head and neck, 46.14–51.89 in the chest and breast group, and 46.60–53.10 in the abdominal and pelvic group. Secondly, although demoralization level changes at different time points during the 3-month study period were observed, these changes seldom reached the statistically significant level. However, it is worth mentioning that the trends of fluctuated demoralization level showed similar patterns among all the cancer patients in different groups. Moreover, as Fig. 1 reveals, in addition to the similar fluctuated demoralization patterns, the demoralization level seems to have a tendency to maintain its equilibrium, that is, the mean scores of the DS-MV at the end of the study tended to approach the baseline mean scores evaluated before RT among all the 3 groups. Thirdly, according to the results of the multivariate GEE analyses, although the participants in the chest and breast cancer group revealed statistically significant changes of demoralization level at 2 time points: the second week after receiving RT and the first month after completing RT, no demographic variables could be considered as a potential factor associated with the changes of the demoralization level among the chest and breast cancer patients at different time points.

Stress evaluation may be affected by cultural differences and sometimes cannot show the actual stress an individual with specific cultural background is burdened with [22]. A previous study has shown that compared to non-Hispanic Whites, Asians are more depressed and anxious and under higher pressure [23]. In Taiwan, the cultural background tends to drive cancer survivors to hide their diseases to avoid humiliations such as loss of dignity and social isolation [24], which may lead the patients to suffer psychological disorders. In fact, multiple studies have shown that patients with cancer in Taiwan tend to be demoralized in general [10, 17, 25, 26]. For example, a study showed the prevalence of demoralization of cancer patient in Taiwan was 49.1% [10], while in comparison it was 37.0% in Australia [18] and 39.2% in Germany [9]. Although this comparison may be subject to bias caused by recruiting participants from different clinical settings, the cross-sectional study design, or the various cancer types, stages, and interventions, the results are consistent with those of the current study, i.e., patients with cancer in Taiwan exhibited an unusually elevated prevalence of high demoralization. In addition to the elevated prevalence of high demoralization, other essential findings were the extremely high DS-MV mean scores and that these scores appeared to maintain a balance even after a period of fluctuation possibly caused by changes in patient’s morale as they went through the treatment that gave them hope and also caused them pain, sometimes combined with potential environmental changes. Based on the foundation of the demoralization concept, the causes of the higher prevalence of high demoralization and the unusually high demoralization level in our study could not be easily and appropriately explained [27]. We will not jump into any quick conclusion, but hypothesize that a complex of social, cultural and cognitive factors may have contributed to these results [28]. Meanwhile, the validity of the DM-MV was evaluated in Taiwan as early as 2010 [8]. The situation of the demoralization levels was consistent with previous studies [29].

The important findings that elucidate the fluctuation of demoralization are as follows: (1) the changes in demoralization did not reach statistically significant levels, and (2) the DS-MV mean scores at the end of study exhibited a trend of approaching the baseline demoralization level. In other words, our findings highlighted the constancy of demoralization rather than fluctuation. A comparison of the diagnostic tests between depressive disorder and demoralization yielded the following findings: (1) The criteria for a major depressive episode, based on the Diagnostic and Statistical Manual of Mental Disorders, Fifth Edition, focused more on the symptoms such as mood status, appetite, sleep quality, fatigue, psychomotor retardation or agitation, and suicidal ideation; and (2) The items of the DS-MV emphasized more developmental, historical, and existential concerns such as the meaning of life, personal value, self-control, spiritual peace, regret, reasons for endurance, and relationships with significant others. In brief, based on the foundation of demoralization, for a living being, the conditions for existence could not be taken for granted. In other words, although we evaluated the demoralization level by using DS-MV at different time points, the scores of many items in the DS-MV might depend on patient’s traits rather than their specific states at the time points. Therefore, based on the current study results, we hypothesize that the baseline range of the demoralization level exists in each patient with cancer, which is decided mainly by the patient's traits and not by the environment the patient is in. That is also to say, the patients had been vulnerable all along, and the changes in the DS-MV scores were only temporary fluctuations while their perception of the meaning, value and purpose of life did not change fundamentally during RT for the newly diagnosed cancer. However, although we inferred that the baseline range of the demoralization level might exhibit a trend of maintaining constancy, the total observation period in our study was less than six months. A previous study has reported that the longer the survival duration of patients with cancer is, the lower the demoralization level is with time [11], which is contrary to our conclusion. Thus, a longer follow-up period is warranted to validate our findings.

### Study limitations

Several limitations of the current study must be discussed. First, the data were collected in 2014, which may not be fully representative of today’s patient population. Second, because demoralization is derived from psychosocial aspects, which is particularly true for patients who experience existential distress, it is appropriate to adopt a mixed-method design with quantitative data and qualitative interviews. This study investigated demoralization of three groups of patients with cancer in relation to the treatment timing, sex, age, education, marital status, income source, employment status, tumor stage, surgery and chemotherapy. We will keep following up on the patients for the effects of clinical outcome and medical cost.

## Conclusion

Overall, the longitudinal study design and analysis with the relevant variables considered, such as cancer regions, cancer stages, and treatment strategies, strengthened the results of our study to provide reference for healthcare professionals when caring for demoralized patients. A consensus regarding the definition of demoralization has gradually been reached, and the principle of treatment strategies such as the whole-person-centered approach has been confirmed. However, in clinical practice, lack of differentiation between demoralization and depression and consequent administration of ineffective therapy are still frequently observed. In this case, the importance of recognizing demoralization phenomena is not only to enable appropriate interventions but to reduce the ineffective care or harmful treatment strategies in patients with cancer as well.

Fluctuations in the demoralization level of cancer patients on RT were observed in our study, but our further analysis showed that the fluctuations were likely temporary and due to patients’ emotional and psychological changes with the treatment, while the demoralization level was actually rather constant and close to the baseline level. Therefore, it is important to avoid rushing to choose interventions for the patients, but to focus instead on carefully differentiating between high demoralization and depressive disorder.

## Data Availability

The datasets generated and/or analyzed during the current study are not publicly available because the dataset was protected by the Institutional Review Board of Kaohsiung Veterans General Hospital, but are available from the first author Pei-Ling Tang on reasonable request.

## Abbreviations

DS: Demoralization scale
DS-MV: Demoralization scale-mandarin version
GEE: Generalized estimating equation
RT: Radiotherapy
SD: Standard deviation
